# Progress in Surgery

**Published:** 1900-03-24

**Authors:** 


### PROGRESS IN SURGERY.

### SURGERY OF THE INTESTINES.

(*Continued from page* 399.)

**Volvulus.**—Rontier[8] operated on a woman, aged 47, suffering from acute obstruction of five days' duration, caused by a volvulus of the sigmoid flexure. After the volvulus was completely untwisted, air rushed into the rectum. On gentle pressure upon the sigmoid flatus escaped from the rectum with a loud noise, followed by abundance of liquid fæces. The lower bowel was then washed out, the gut carefully reduced, and the abdomen closed. Recovery was rapid. Sacquépée[9] reports a fatal case of volvulus of the ileum from bands after appendicitis. The vermiform appendix, which was very thick, ran astride the end of the ileum, to which it adhered firmly. In separating a dense band the ileum was torn, and the patient died on the eighth day.

**Intestinal Obstruction**, as a result of taxis for a right femoral hernia six months previously, is reported by Mollard and Bernay[10] as occurring in a woman aged 44. The taxis had apparently resulted in adhesions and stricture of the ileum. Enteroplasty—after the manner of pyloroplasty—was performed, and the patient made a good recovery.

**Intestinal Resection and Anastomosis.**—Morisani[11] is convinced that the good results of a bowel suture are not dependent upon the complexity, nor the strength, nor the number of layers, but rather upon the certainty of protecting the thread from infection from the contents of the intestinal canal. He recommends the following procedure which belongs to the class of intestinal anastomoses by invagination and resection of mucous membrane: Following the required resection, the mucous membrane of the end of the intestine to receive the invaginating portion is grasped with forceps, incised vertically for from four to six centimetres, and a circular flap of mucous membrane of this breadth removed. The other end of the intestine is now invaginated for a distance of several centimetres into the lumen thus prepared. The two ends are held in apposition by two or three fixation sutures, and a continuous suture of silk is applied round the gut in such a manner as to securely fix the entire denuded surface of the invaginating to the corresponding serous surface of the invaginated end. This suture begins at the mesenteric border of the invaginated bowel. The needle pierces the bowel wall, and, entering the muscular coat of the invaginated bowel, it traverses this muscular coat to the level of the free edge of the denuded portion of the invaginating bowel. The needle is brought outside at this point, and this, the first stitch of the continuous suture, is tied, the end being left long. This over and over stitch is continued around the entire circumference of the bowel—the stitches being placed somewhat obliquely. When the mesenteric border is again reached the last stitch is secured by tying it to the end of the first, which was left long for the purpose. The remainder of the operation is completed in the usual manner. The chief objection to this and similar operations is the danger of post-operative progression of the invagination, sloughing of the ends, stricture at the site of union, and the difficulty in differentiating the distal from the proximal end. Barker[12] thinks that with the improved methods of intestinal anastomosis gangrenous intestine in cases of hernia are best treated by primary resection. The factors which contribute most to a successful result are (*a*) the preliminary treatment, (*b*) the details of the operation, and (*c*) the after treatment. In all cases where there has been stercoraceous vomiting the stomach should be washed out with a tube and funnel with warm brandy and water until quite clean. Hypodermic transfusion is most valuable in making up for the fluid lost by vomiting, sweating, and lung exhalation, and it is well to give a copious enema of warm brandy and water. In the operation everything which saves time is of the utmost importance. The bowel is emptied by pressure, and clamped. For this purpose nothing is better than Doyen's forceps, the long blades of which should run close to the mesenteric attachment, and across the two lumina of the afferent and efferent tubes first placed accurately side by side, the smaller efferent tube being spread out as much as possible to correspond with the larger afferent. The loop is then well wrapped in sterile gauze, and removed by scissors cutting along the mesenteric border and obliquely across the two tubes, so that the free margin of what is left is shorter than the attached. The ends are then united without removing the forceps. The hypodermic transfusion of saline solution twice a day to the extent of a pint or so each time is an important factor in after treatment. Nutritive enemata every four hours, and hot water enemata every four hours, alternating with the last form a routine treatment in all cases. In excision of the ascending colon for carcinoma Eccles[13] thinks that there is a very considerable danger in attempting to perform the ideal operation, namely, that of primary excision and anastomosis; and if there is complete obstruction such procedure would seem hardly justifiable. In such cases it is wiser to merely bring the intestine out on the abdominal wall and make an artificial anus—the growth being excised at a subsequent operation. In a case of gangrenous hernia Dreesmann[14] successfully resected no fewer than seven feet of ileum.

**Surgical Treatment of Typhoid Fever.**—Armstrong[15] has operated ten times for typhoid perforation of the intestine with but one recovery. In all of them the operation had been too long delayed, as, on opening the abdomen, gas and fæcal matter escaped, and it was quite evident that a very considerable portion of the peritoneal cavity had been infected. Sacquépée[16] also records an unsuccessful case, which was performed within fifteen hours of the onset of symptoms. Ryan[17] thinks that twelve hours after the occurrence of the lesion is the most favourable time for operating; when the patient has rallied from the shock, and before general septic peritonitis has set in.

[8] Brit. Med. Jour., Dec. 30, 1899, Epitome No. 499. [9] Ibid., Sept. 23, 1899, Epitome No. 243. [10] Ibid., Oct. 14, 1899, Epitome No. 295. [11] Annals of Surg., Dec. 1899, p. 763. [12] Lancet, Dec. 23, 1899, p. 1729. [13] Brit. Med. Jour., Jan. 13, 1900, p. 69. [14] Ibid., Sept. 9, 1899, Epitome No. 191; and Berl. Klin. Woch, No. 16, 1899. [15] Ibid., Oct. 7 1899, Epitome No. 279. [16] Ibid., Oct. 14, 1899, Epitome No. 299 [17] Austral. Med. Gaz., Aug. 21, 1899, p. 337.

